# Advancing cellular insights: Super-resolution STORM imaging of cytoskeletal structures in human stem and cancer cells

**DOI:** 10.1016/j.bbrep.2024.101798

**Published:** 2024-07-25

**Authors:** Anupam Bharadwaj, Amalesh Kumar, Sam Padalumavunkal Mathew, Rumela Mitra, Jina Bhattacharyya, Bithiah Grace Jaganathan, Bosanta R. Boruah

**Affiliations:** a Department of Physics, Indian Institute of Technology Guwahati, Guwahati, 781039, Assam, India; b Department of Biosciences and Bioengineering, Indian Institute of Technology Guwahati, Guwahati, 781039, Assam, India; c Department of Haematology, Gauhati Medical College, Guwahati, 781032, Assam, India; d Jyoti and Bhupat Mehta School of Health Sciences and Technology, Indian Institute of Technology Guwahati, Guwahati, 781039, Assam, India

**Keywords:** Optical super-resolution microscopy, Stochastic optical reconstruction microscopy, actin cytoskeleton, Tubulin, β-catenin, Breast cancer

## Abstract

Fluorescence microscopy is an important tool for cell biology and cancer research. Present-day approach of implementing advanced optical microscopy methods combined with immunofluorescence labelling of specific proteins in cells is now able to deliver optical super-resolution up to ∼25 nm. Here we perform super-resolved imaging using standard immunostaining protocol combined with easy stochastic optical reconstruction microscopy (easySTORM) to observe structural differences of two cytoskeleton elements, actin and tubulin in three different cell types namely human bone marrow-derived mesenchymal stem cells (MSCs), human glioblastoma (U87MG) and breast cancer (MDAMB-231) cells. The average width of the actin bundle obtained from STORM images of stem cells is observed to be larger than the same for U87MG and MDAMB-231 cells. No significant difference is however noticed in the width of the tubulin within the same cells. We also study the functional effect on the 2D migration potential of MDAMB-231 cells silenced for NICD1 and β-catenin. Although similar migration speed is observed for cells with the above two conditions compared to their control cells, easySTORM images show that widths of the actin in MDAMB-231 cells in β-catenin silenced is significantly lower than the same in control cells. Such minute differences however are not observable in widefield images. The outcome of our easySTORM investigation should benefit the researchers carrying out detailed investigations of the cellular structure and potential therapeutic applications.

## Highlights

• Super-resolution based on easySTORM of MSC, U87MG and MDAMB-231 cell lines.
• Actin bundle width in MSC appears larger than in U87MG and MDAMB-231 cells.
• Estimated minimum width of actin bundles in the breast cancer cell line is ∼32 nm.
• Tubulin in glioblastoma and breast cancer cell lines shows minimum width as 24 nm.
• easySTORM shows a ∼25 % decrease in actin bundle width in β-catenin silenced cells.

## 1 Introduction

Fluorescence microscopy has been an important tool in cell biology to study the architecture and dynamics of cellular and tissue components. Comprehensive analysis of structural changes in biological systems is however restricted in diffraction limited optical microscopy, where the best achievable resolution is ∼200 nm.

In recent years, advanced fluorescence microscopy techniques are being developed to allow super-resolved imaging of fixed, wet and live cells and tissues up to 25 nm of resolution [1]. Point scanning based stimulated emission depletion microscopy (STED) [2] and widefield based single molecule localization microscopy methods such as stochastic optical reconstruction microscopy (STORM) [3] are the popular and widely accepted optical super-resolution microscopy techniques. These techniques provide resolution of typically 20–50 nm subject to the availability of the suitable fluorophores and the laser. However, easySTORM [4], a variation of STORM has a simpler setup that can be implemented more easily and that reduces the overall cost of the system compared to STED. STORM is based on reconstructing super-resolved images of precisely localized fluorophore probes by recording thousands of images when the fluorophores undergo random on/off blinking between emissive and dark states in a favorable chemical environment under a suitable source of excitation [5].

In this work, we utilize a modular research grade optical microscope based on the *openFrame* concept [6] and demonstrate the implementation of sub-diffraction optical fluorescence imaging with easySTORM for studying cytoskeleton elements in cancer and normal cells.

Actin and tubulin filaments are two essential cytoskeleton elements that provide cell shape, facilitate cell migration, movement of organelles and protein within the cells, and mediate cell division. Though there are reports [7,8] on optical super-resolution imaging of actin filaments and tubulin using STORM, there lacks adequate study on the structural distinction between the cytoskeleton elements in human normal and cancer cells using a super-resolution imaging technique such as easySTORM. In this paper we perform easySTORM imaging of actin and tubulin prepared using direct and indirect immunostaining methods for three different cell types, namely: human bone marrow derived mesenchymal stem cells (MSCs), human glioblastoma cell line U87MG and human breast cancer cell line (MDAMB-231). The average width of both actin and tubulin is estimated from the easySTORM images.

It is worth noting that Wnt/β-catenin and Notch signalling pathways regulate cell functions and contribute to a range of cell fate decisions in neuronal, cardiac, immune, endocrine and breast tissue development. Notch signalling and Wnt/β-catenin signalling are also known to modulate the actin arrangement within the cells and are vital for cell motility and metastasis [9,10]. Here we first study the 2D cell migration potential under brightfield illumination to investigate the effect of NICD1 (Notch1 Intracellular Domain) and β-catenin silencing in the migration speed of the MDAMB-231 cells with respect to their control cells. The results obtained from these investigations have prompted us to further apply easySTORM to perform a comprehensive analysis of the average widths of actin in the MDAMB-231 cells under NICD1 and β-catenin silenced conditions. This study helps reveal the arrangement and the width of actin bundles upon silencing of NICD1 and β-catenin in triple negative breast cancer cells, MDAMB-231. Actin arrangement is indispensable to the migration and invasion of cancer cells. Therefore, studying the arrangements of actin upon treatment with various agents helps provide useful insights into the metastatic ability of the cancer cells to migrate and invade surrounding tissues.

## 2 Materials and methods

### 2.1 Experimental arrangement

**Fig. 1:**
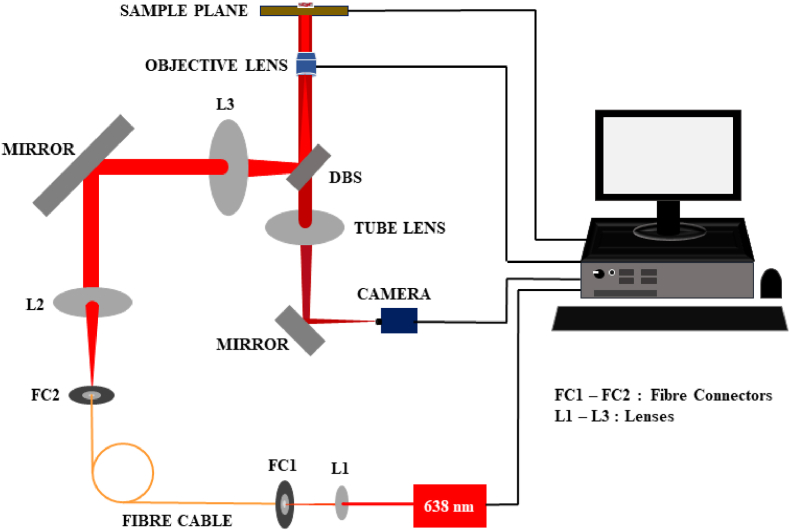
A schematic of the experimental arrangement for widefield and easySTORM.

As mentioned already, the experimental arrangement comprises the *openFrame*-based modular research-grade optical microscope [6], developed in a cost-effective way and operated in the widefield epi-fluorescence mode. As seen in Fig. 1, the excitation beam from the 638 nm laser is coupled to the fibre connector, FC1, via lens, L1 to be transmitted through a fibre cable. At the other end a fibre connector, FC2, is positioned at the focus of the L2 to render a collimated beam that is incident on a mirror. The lens L3 then, after reflection from the dichroic beam splitter (DBS) cube, focuses the beam at the back focal plane of the objective lens to get Koehler illumination on the sample plane. The resulting fluorescent emission from the sample passes through the DBS cube and proceeds to the camera, after reflection from a mirror followed by focusing by the tube lens.

We use an oil immersion objective lens (PlanF 100× 1.3 NA, Amscope) and a fan-cooled CMOS monochrome camera with 5440x3648 pixels. The DBS cube contains a multi-line dichroic beam splitter with a matching emission filter. The camera and the movement of the sample plane and the Z stage holding the objective lens are controlled with the help of an open-source μManager software via a PC interface. The power of the excitation laser can be controlled by the same software. Additionally, we have used a Zeiss (Axio Vert.A1) optical microscope employing the N-Achroplan 5X objective lens for brightfield imaging.

### 2.2 Image acquisition and reconstruction

The process of imaging starts with acquiring of a widefield image using the minimum available excitation intensity (0.2 kW cm^-2^) of the excitation laser. We then increase the excitation intensity to 0.7 kW cm^-2^ to activate fluorescence blinking [5] and acquire about 15,000, 20,000 and 7000 image frames at the rate of 33 frames per second (fps) for actin, tubulin and actin of cells with cellular conditions, respectively. Post-acquisition and reconstruction are conducted employing the ThunderSTORM plugin inside the open-source image analysis software Fiji [11]. During the post-acquisition process, the stack of images is rendered at a pixel size of 21.6 nm with an Analog to Digital Unit (ADU) conversion factor 4 e^−^. Additional parameters in ThunderSTORM, such as wavelet filter as the image filtering method, Gaussian using the maximum likelihood function as the fitting algorithm and Average Shifted Histogram with a 10X magnification for visualization, are employed for image reconstruction. Post-reconstruction includes the drift analysis using the inbuilt drift correction method in ThunderSTORM for the final display of the image.

The resolution characteristics of our *openFrame*-based microscope was measured by imaging isolated Carboxyl Quantum Dots (QDs) (size ∼ 20 nm) as reported in our previous work [6] and the width of the lateral point spread function was found to be 324 ± 3 nm.

### 2.3 Sample preparation

As mentioned earlier in section 1, sample preparation involves direct and indirect immunostaining methods for staining actin and tubulin for the MSCs, U87MG and MDAMB-231 cells. The subsequent sections discuss the detailed sample preparation methods.

#### 2.3.1 Isolation and immunostaining staining of MSCs

Bone marrow aspirates were obtained from Gauhati Medical College Hospital, following protocols approved by the local ethical committee and after informed consent from the patients included in the study. Following previously established protocols [12], the bone marrow mesenchymal stem cells (MSCs) were isolated. MSCs were seeded at a density of 2000 cells/cm^2^ onto glass coverslips. Subsequently, the cells were allowed to attach and multiply inside a humidified incubator of 5 % CO_2_ for 24 hr. The cells were washed twice with pre-warmed (37 °C) phosphate-buffered saline (PBS) and fixed using 4 % (v/v) formaldehyde (Merck, Cat. no. 1.94989.0521) for 15 min at 4 °C. The cells were permeabilized using 0.1 % (v/v) Triton X-100 (Merck, Cat. no. 1086031000) for 15 min at room temperature. Blocking was done with 2 % (w/v) fetal bovine serum (FBS; Invitrogen) in PBS and actin was stained with Alexa Fluor™ 647-conjugated Phalloidin (Invitrogen, Cat.no. A22287). In case of tubulin staining, the cells were stained with antibody against β-tubulin (Cell Signaling Technology, Cat. No. 2146) and incubated overnight at 4 °C. The cells were stained with Alexa Fluor 647 tagged secondary antibody (Invitrogen, Cat. No. A31573). The coverslips were mounted onto glass slides using polyvinyl alcohol (PVA) based Mowiol (Sigma) as the imaging buffer containing 1 % β-mercaptoethanol (β-ME) (Himedia) to facilitate the on/off blinking [7], and allowed to dry for 36–48 hr in a 37 °C oven. Finally, the coverslips were sealed with transparent nail varnish before proceeding with STORM imaging.

#### 2.3.2 Immunostaining of U87MG and MDAMB-231 cells

The U87MG and MDAMB-231 cells were acquired from the National Centre for Cell Science (NCCS), Pune, India. The cells were cultured in high-glucose Dulbecco's modified Eagle's medium (DMEM, Gibco, Cat. no. 12800-058) supplemented with 10 % FBS (Gibco). The cells were seeded at a density of 2000 cells/cm^2^ onto sterile glass coverslips, and the cells were allowed to attach for 24 h. The cells were attained with Phalloidin-Alexa Fluor™ 647 or anti-β-tubulin as described earlier for MSCs.

#### 2.3.3 Silencing of β-catenin and NICD1

MDAMB-231 cells were transduced with lentiviral vectors containing shRNA for NICD1 (Notch 1 Intracellular Domain) and β-catenin (Sigma). As control, the cells were transduced with lentiviral vector containing the scramble sequence. Lentiviral transduction was done as reported previously [13]. Briefly, 293T HEK cells (Invitrogen) were transfected with pLKO.1-Puro plasmid containing the shRNA or scramble sequence along with the packaging plasmids (pMD2.G and psPAX2) using polyethyleneimine (PEI) method. Viral supernatant was collected after 48 hr and 72 hr after transfection and the target cells were transduced in the presence of polybrene (4 μg/mL, Sigma). The cells were kept under puromycin (2 μg/mL) for 4 days to select the transduced cells.

#### 2.3.4 Scratch wound assay (migration)

25000 cells/cm^2^ were seeded in triplicates in a 24-well plate and allowed to grow until they formed a monolayer. This was followed by serum starvation for a duration of 12–16 h. A scratch, using a microtip, was made and 2 % FBS supplemented media was added to each well. The migration speed was determined by taking images of cells at 4-hr intervals, identifying open area using T-scratch software followed by calculation of the distance migrated at each time point (4 hr, 8 hr and 12 hr) in μm/hour.

## 3 Results

We performed widefield epi-fluorescence and easySTORM imaging of actin and tubulin filaments in MSCs, U87MG and MDAMB-231 cells using our modular research grade optical microscope discussed in section 2.1. Sample preparation, as discussed in section 2.3, involved direct and indirect immunostaining of the actin and tubulin with Alexa Fluor 647 (AF-647) dyes. Super-resolved images are reconstructed employing steps as discussed in section 2.2.

### 3.1 Imaging of actin

**Fig. 2:**
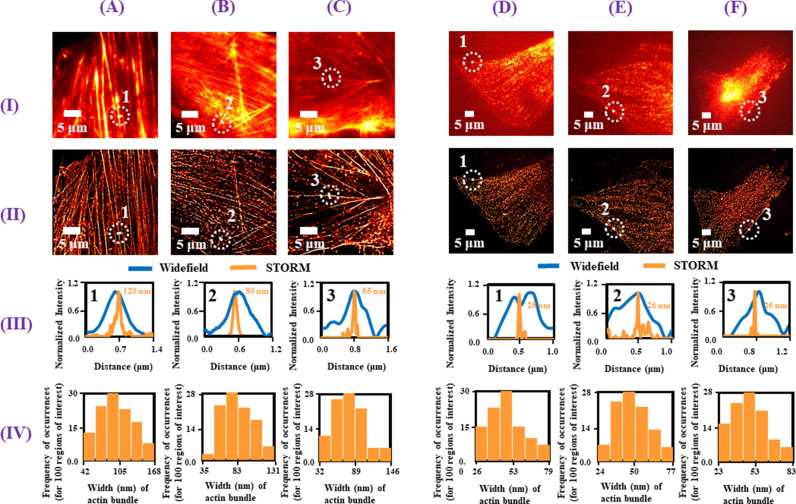
Columns (A), (B), and (C) correspond to results for actin in MSCs, U87MG and MDAMB-231 cells, respectively, while columns (D), (E), and (F) correspond to results for tubulin in same cells. (I) shows the widefield images while (II) shows the respective super-resolved images obtained using easySTORM. (III) shows the combined line plots of the regions of interest 1, 2 and 3 chosen from the widefield and the STORM images of actin and tubulin respectively. The line plots of widefield and STORM are colored blue and orange respectively. (IV) shows the histogram plots representing the frequency of occurrences of the width of actin bundle and tubulin respectively chosen in each easySTORM image. 100 regions of interest are selected from each image for the histogram plot. Values are measured as mean + SD. For actin, MSCs compared to U87MG (p < 0.0001) and MDA-MB-231 (p < 0.0001) cells.

In Fig. 2 (I) (A), (B) and (C), the widefield images (pseudo-colored in red) of actin filaments chosen from three fields of views (FOVs) are shown for the MSCs, U87MG cells and MDAMB-231 cells, respectively. Fig. 2 (II) (A), (B) and (C) are the corresponding reconstructed super-resolved images acquired using STORM (pseudo-colored in red). In Fig. 2 (III) (A), (B) and (C), we show the combined line plots of the ROIs 1, 2 and 3 chosen at equivalent positions in the widefield and the STORM images. The full width at half maximum (FWHM) of the line plots for the reconstructed images give widths of 120 nm, 80 nm and 65 nm for the ROIs 1, 2 and 3 respectively. On the other hand, the actin filaments are not clearly observed in the widefield images.

To better interpret the results, we randomly select 100 different regions of interest (ROIs) from each widefield image in Fig. 2 (I) (A), (B) and (C). The average widths of the actin calculated from the FWHMs of the line plots drawn over these ROIs are found to vary approximately between 300 and 700 nm. The same ROIs are then selected in their corresponding STORM images. The average widths of the actin from the reconstructed images are estimated as 101 ± 30 nm for MSCs, 81 ± 20 nm for U87MG cells and 77 ± 23 nm for MDAMB-231 cells. A graphical representation of these findings is shown in Fig. 2 (IV) (A), (B) and (C) where we show the histogram plots for 100 measurements from each of the STORM images. The maximum frequency of occurrences falls in the range of 84–105 nm, 67–83 nm and 70–89 nm for the MSCs, U87MG and MDAMB-231 cells, respectively. We detect significantly thicker actin bundles for MSCs compared to U87MG (p < 0.0001) and MDA-MB-231 (p < 0.0001) cells. The minimum widths of the actin from the FWHM calculations are found to be approximately 42 nm, 35 nm and 32 nm for the MSCs, U87MG and MDAMB-231 cells, respectively. However, we also measure the width of actin for 5–10 cells in each cell type and the results do not show a significant difference.

To the best of our knowledge such detailed investigation of actin has not been reported so far for these specific cells using optical microscopy. Using electron microscopy, the cross-sectional diameter of an actin filament was found to be approximately 7 nm [14] and it was further observed that the actin filaments with specific binding proteins group together within a cell to form bundles, where each bundle consists of a few to thousands of actin filaments [15]. Therefore, our findings with easySTORM suggest that actin bundles in MSCs, U87MG cells and MDAMB-231 cells may be due to the grouping of different numbers of actin filaments. Further according to the analysis using cryo-electron microscopy, the interspacing of two parallel bound actin filaments may vary from 11 to 36 nm [15] depending on the structural arrangement of the type of protein binding in the two actin filaments. Hence, using the interspacing between two actin filaments as given by electron microscopy and the widths of the actin bundles as estimated from our easySTORM images, the number of actin filaments per bundle is found to vary approximately between (3–6), (2–5) and (2–5) for MSCs, U87MG and MDAMB-231 cells respectively.

### 3.2 Imaging of tubulin

In Fig. 2 (I) (D), (E) and (F), we show the widefield images (pseudo-colored in red) of tubulin from three different FOVs in the MSCs, U87MG and MDAMB-231 cells, respectively, mounted in three different microscope slides. Fig. 2 (II) (D), (E) and (F) are the corresponding super-resolved images acquired using STORM (pseudo-colored in red). The line plots of the ROIs 1, 2 and 3, chosen in the widefield images and the corresponding STORM images are seen in Fig. 2 (III) (D), (E) and (F). The widths of the tubulin, from the FWHM of these line plots corresponding to the STORM images, are measured as 28 nm, 26 nm and 26 nm for MSCs, U87MG and MDAMB-231 cells, respectively.

Here, we again chose 100s of ROIs randomly in each widefield image. The average widths of tubulin for the three cells measured from the FWHM of the line plots drawn over these ROIs approximately range between 300 and 700 nm. However, from the reconstructed super-resolved images, the average widths for the same 100 ROIs are measured to be 51 ± 15 nm for MSCs, 48 ± 11 nm for U87MG cells and 48 ± 13 nm for the MDAMB-231. The results closely match the diameter range of 25 nm–51 nm for tubulin as reported previously using electron microscopy [16,17]. Fig. 2 (IV) (D), (E) and (F) shows the histogram plots for the frequency of occurrences of the 100 measurements from each of the STORM images. The maximum frequency of occurrences lies in the range 44–53 nm, 42–50 nm and 43–53 nm for MSCs, U87MG and MDAMB-231, respectively. Furthermore, we assess the tubulin width in 5–10 cells of each type, and the results revealed no significant difference. Additionally, the minimum widths of tubulin measured from the super-resolved images are approximately 26 nm, 24 nm and 24 nm respectively, for MSCs, U87MG cells and MDAMB-231, which closely match the minimum outer diameter range of 23–27 nm for tubulin as reported earlier using electron microscopy [18].

### 3.3 Study on NICD1 and β-catenin silenced in MDAMB-231 cells

#### 3.3.1 Analysis of migration potential through scratch wound healing assay

**Fig. 3:**
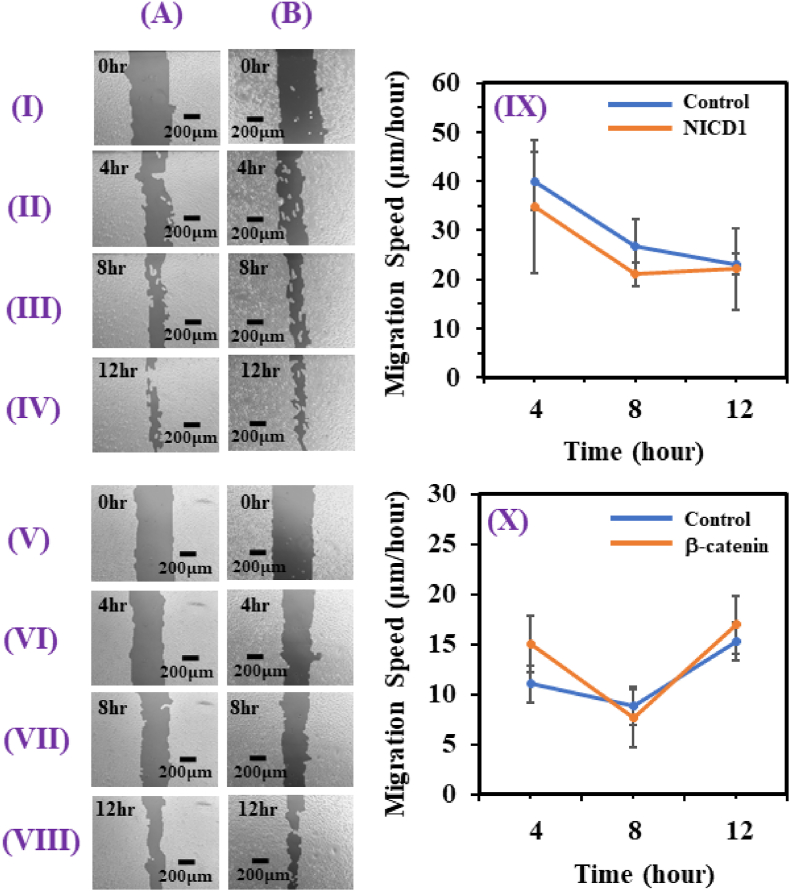
(I) - (IV) are the brightfield images of (A) MBAMB-231 control cells and (B) NICD1 silenced MBAMB-231 cells, at 0, 4, 8 and 12 hr, respectively. (V) - (VIII) are the brightfield images of (A) control cells and (B) β-catenin silenced cells at 0, 4, 8 and 12 hr, respectively. (IX) and (X) display the migration speed versus time plots of the NICD1 with its control (p = 0.12) and β-catenin silenced with its control (p = 0.44), respectively.

Migration of cancer cells is key to their ability to metastasize to distant sites. To test the migratory ability of the MDAMB-231 cells upon silencing of NICD1 and β-catenin- mediated signaling, a scratch wound healing assay is performed for these conditions and compared with their respective control cells. Fig. 3 (I) - (IV) (A) are the brightfield images of the control cells as observed at 0, 4, 8 and 12 hr respectively, while Fig. 3 (I) - (IV) (B) represent the brightfield images of the cells as observed at 0, 4, 8 and 12 hr respectively for the NICD1 silencing. The migration speed (μm ⁄ hour) of NICD1 silenced cells while being lesser than the control is found not to be statistically significant (p = 0.12) as shown in Fig. 3 (IX). We have performed a similar experiment with the β-catenin silenced cells as shown in Fig. 3 (V) - (VIII) (B) at 0, 4, 8 and 12 hr respectively and compared the results with the respective control cells as shown in Fig. 3 (V) - (VIII) (A) at 0, 4, 8 and 12 hr respectively. The change in the migration speed with respect to time for β-catenin silenced and control cells does not seem to vary significantly (p = 0.44) as shown in Fig. 3 (X).

#### 3.3.2 Imaging of actin bundle distribution

**Fig. 4:**
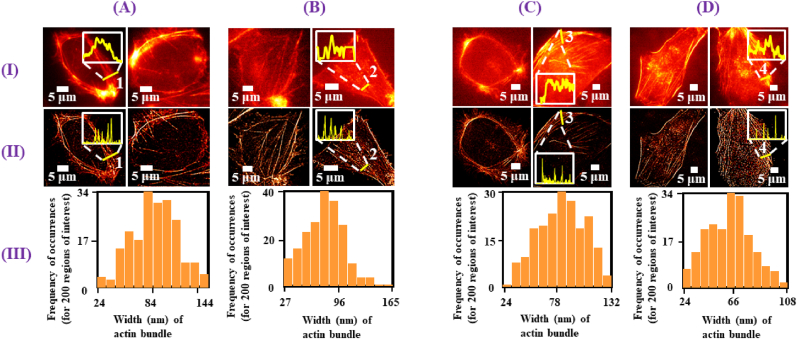
Columns (A) and (B) correspond to results in control and NICD1 silenced MDAMB**-**231 cells, respectively while columns (C) and (D) correspond to results in control and β-catenin silenced MDAMB**-**231 cells, respectively. (I) shows the widefield images while (II) shows their respective super-resolved images reconstructed using easySTORM. (III) is the histogram plots representing the frequency of occurrences of the width of actin for 200 regions of interest randomly chosen from the easySTORM images (measured as mean + SD), p < 0.0001 for control versus β-catenin silenced MDAMB-231 cells. The insets in (I) and (II) show intensity line plots (colored yellow) of the regions of interest marked as 1, 2, 3 and 4.

We then perform easySTORM of actin bundle in control, NICD1 silenced and β-catenin silenced MDAMB-231 cells. Fig. 4 (I) (A) and (B) represent the widefield images in control and NICD1 silenced MDAMB-231 cells, while Fig. 4 (II) (A) and (B) are the corresponding STORM images. Intensity line plots drawn over the ROIs 1 and 2 and seen in the insets indicate that the actin bundles are better resolved in the STORM images relative to the widefield images.

We measure the width of the actin bundle from the widefield images and STORM images for the two conditions. The data are collected by selecting 200 regions of interest of single actin bundle in different FOVs. The average width measured from the widefield images ranges between 300 and 800 nm. However, from the reconstructed images the average widths are measured as 83 ± 23 nm and 79 ± 24 nm for control and NICD1 silenced cells, respectively. Thus, the silencing of NICD1 in MDAMB-231 cells has a limited impact on the dimensions of the actin bundle in terms of average width. From the histogram plots as shown in Fig. 4 (III) (A) and (B) we can see the maximum frequency of occurrence lies in the range 74–84 nm and 73–85 nm for control and NICD1 silenced cells, respectively.

We also extended our analysis to observe the changes in the actin arrangement and bundle width in MDAMB-231 cells when β-catenin was downregulated by gene silencing. The widefield images for the control and β-catenin silenced cells are shown in Fig. 4 (I) (C) and (D), respectively, while the corresponding easySTORM images are shown in Fig. 4 (II) (C) and (D), respectively. Intensity line plots drawn over the ROIs 3 and 4 and seen in the insets indicate that the actin bundles are again better resolved in the STORM images relative to the widefield images.

We also measured the average widths of the actin bundle from the easySTORM images by selecting 200 ROIs of single actin bundle in different FOVs. We estimate the average widths as 84 ± 23 nm and 63 ± 18 nm for the control and β-catenin silenced conditions, respectively. Thus, we observe a ∼25 % decrease in the average width of actin bundle in the β-catenin silenced cells (p < 0.0001) compared to the control MDAMB-231 cells. The histogram plots of 200 measurements for each condition are shown in Fig. 4 (III) (C) and (D), where we observe that the highest frequency of occurrences lies between 78-86 nm and 59–66 nm for the control and β-catenin silenced conditions, respectively.

## 4 Discussion

Our experiments have demonstrated the implementation of super-resolved fluorescence microscopy using easySTORM to image three types of cells, namely, human bone marrow derived mesenchymal stem cells (MSCs), U87MG (human glioblastoma cells) and MDAMB-231 (human breast cancer cells) in terms of their two cytoskeleton elements, actin and tubulin. To the best of our knowledge a comparative study of the actin and tubulin widths in the above cells using easySTORM has not yet been reported.

As indicated in Table 1, a significant difference is noticed in the average width of the actin bundles of the three cell types. MSCs show significantly thicker actin bundles compared to U87MG and MDAMB-231 cells. We find that the width of the actin bundle is significantly small in MDAMB-231 cells (∼32 nm) compared to the other cell types and in the case of tubulin, U87MG and MDAMB-231 cells showed the minimum width of ∼24 nm. However, no significant difference is noticed in the average width of the tubulin in these cells. Additionally, we have estimated the average number of actin filaments per bundle taking the interspacing between two actin filaments as given by electron microscopy [14] and the widths of the actin bundles as estimated from our easySTORM images. The number of actin filaments per bundle is found to vary approximately between (3–6), (2–5) and (2–5) for MSCs, U87MG and MDAMB-231 cells, respectively.

**Table 1:** Average and minimum widths of actin bundle and tubulin from FWHM measurements in easySTORM images of MSCs, U87MG cells and MDAMB-231 cells.

Cell Name	Average width from FWHM measurements using easySTORM (average of 100 ROIs)		Minimum width from FWHM measurements using easySTORM	
	ACTIN BUNDLE	TUBULIN	ACTIN BUNDLE	TUBULIN
human bone marrow derived Mesenchymal stem cells (MSCs)	101 ± 30 nm	51 ± 15 nm	42 nm	26 nm
U87MG (glioblastoma cell line)	81 ± 20 nm	48 ± 11 nm	35 nm	24 nm
MDAMB-231 (breast cancer cell line)	77 ± 23 nm	48 ± 13 nm	32 nm	24 nm

We further investigate the 2D cell migration potential of MDAMB-231 cells under β-catenin and NICD1 silenced conditions. From the migration assay analysis, we are unable to notice any significant variation in the migration speed. However, the study with easySTORM confirmed a ∼25 % decrease in actin bundle width in the case of β-catenin silenced cells (63 ± 18 nm) compared to the control cells (84 ± 23 nm), which is not observable under widefield imaging. On the other hand, only a slight difference in the width of the actin bundle between the NICD1 silenced (83 ± 23 nm) and the corresponding control cells (79 ± 24 nm) is observed.

The significant decrease in actin bundle width during β-catenin silencing is an interesting finding that suggests its role in regulating the α-catenin and E-cadherin interaction essential for cancer cell migration and invasion [10]. Changes in actin arrangement represent the alterations in the migration and invasion potential which is an important event during metastasis. Actin is an integral part of the assembly that aids in cell migration by the formation of lamellipodia and filopodia [19,20]. β-catenin binds to α-catenin in its monomeric state. In its homodimer state, α-catenin competes with Arp2/3 complex to bind to actin bundles and prevents actin polymerization [21]. Inhibition of actin polymerization is known to affect the ability of the cells to migrate. In our study, silencing β-catenin resulted in the formation of smaller actin bundles in MDAMB-231 cells. Thus, β-catenin silencing may result in increased availability of α-catenin and a concomitant reduction in actin polymerization as observed by us. Furthermore, understanding the actin width and arrangement will help in determining the migration/invasion ability of cancer cells and our study shows that β-catenin modulates the actin arrangement, and consequently, their invasion potential and the disease prognosis.

To conclude, the present work has reported the standard immunostaining method combined with easySTORM for studying the cellular structures, especially cytoskeleton elements, beyond the diffraction limit of optical microscopy, which can create new avenues, in general, leading to further in-depth study of cellular biology and cancer research.

## Statistical analysis

The variability in the width of actin bundles across different cell types and conditions was quantitatively assessed using one-way ANOVA. p values < 0.01 are considered statistically significant.

## CRediT authorship contribution statement

**Anupam Bharadwaj:** Writing – original draft, Visualization, Methodology, Investigation. **Amalesh Kumar:** Writing – original draft, Visualization, Methodology, Investigation. **Sam Padalumavunkal Mathew:** Methodology, Data curation. **Rumela Mitra:** Methodology, Data curation. **Jina Bhattacharyya:** Data curation, Conceptualization. **Bithiah Grace Jaganathan:** Writing – review & editing, Data curation, Conceptualization. **Bosanta R. Boruah:** Writing – review & editing, Visualization, Methodology.

## Declaration of competing interest

The authors declare that they have no known competing financial interests or personal relationships that could have appeared to influence the work reported in this paper.

## Data Availability

Data will be made available on request.
